# Giant Pheochromocytoma With Non-classical Symptoms: A Case Report to Expand Clinical Awareness

**DOI:** 10.7759/cureus.88445

**Published:** 2025-07-21

**Authors:** Inass Chaari, Ayoub Idrissi, Fouad Hajji, Lhoussaine Abainou, Hamza El Jadi, Azzelarab Meftah, Hicham Baïzri

**Affiliations:** 1 Endocrinology, Diabetes and Metabolism, Avicenna Military Hospital, Marrakech, MAR; 2 Urology, Caddi Ayyad University, Ibn Sina Military Hospital, Marrakech, MAR; 3 Endocrinology and Diabetology, Caddi Ayyad University, Avicenna Military Hospital, Marrakech, MAR

**Keywords:** adrenal glands, catecholamine hypersecretion, giant pheochromocytoma, hypertension, scintigraphy

## Abstract

Pheochromocytomas are rare neuroendocrine tumors arising from chromaffin cells of the adrenal medulla or extra-adrenal paraganglia. Their clinical presentation varies widely among individuals and is primarily linked to the biological effects of excessive catecholamine secretion. We report an unusual case of pheochromocytoma presenting as epigastric heaviness.

A 65-year-old male with a known history of type 2 diabetes mellitus, treated with metformin, and well-controlled hypertension managed with amlodipine, presented with a complaint of persistent epigastric heaviness in the absence of associated symptoms. Abdominal CT imaging revealed a large, locally infiltrative right adrenal mass measuring 92 x 82 x 80 mm. As part of the etiological work-up, serum potassium and urinary cortisol levels were within normal limits. The diagnosis of pheochromocytoma was established based on significantly elevated urinary metanephrines and normetanephrines, exceeding the normal values by 55-fold and 23-fold, respectively. The evaluation for multiple endocrine neoplasia syndromes was negative. Iodine-123 metaiodobenzylguanidine (¹²³I-MIBG) scintigraphy confirmed the presence of a hyperfixating adrenal mass, consistent with pheochromocytoma. Following adequate preoperative pharmacologic preparation, the patient underwent a right adrenalectomy. Histopathological analysis confirmed the diagnosis of pheochromocytoma. The postoperative course was uneventful.

Pheochromocytomas are most often benign but may be associated with severe cardiovascular complications due to catecholamine excess. Their clinical diagnosis remains challenging due to the lack of specific signs and symptoms. The diagnosis relies on biochemical assays of catecholamine metabolites, supported by functional and anatomical imaging techniques. Surgical excision, preceded by meticulous pharmacologic preparation, remains the cornerstone of treatment. Early diagnosis and appropriate management are essential to prevent potentially life-threatening complications and ensure favorable outcomes.

## Introduction

Pheochromocytoma is a neuroendocrine tumor of the adrenal medulla, involving cells that produce catecholamines. It is a rare entity. Some authors report that approximately 10 individuals in 1 million are diagnosed with pheochromocytoma each year [1]. 

Giant pheochromocytomas are typically larger than 7 cm to 10 cm, and their occurrence and presentation are not clearly established. Therefore, the evaluation of every patient with pheochromocytoma should involve a comprehensive assessment of metastatic risk, beginning with the initial clinical evaluation and continuing through biochemical profiling and imaging (including tumor size and location), as well as histological, immunological, and molecular analyses [2]. This case report highlights a giant pheochromocytoma with an atypical clinical manifestation.

## Case presentation

**Figure 1:**
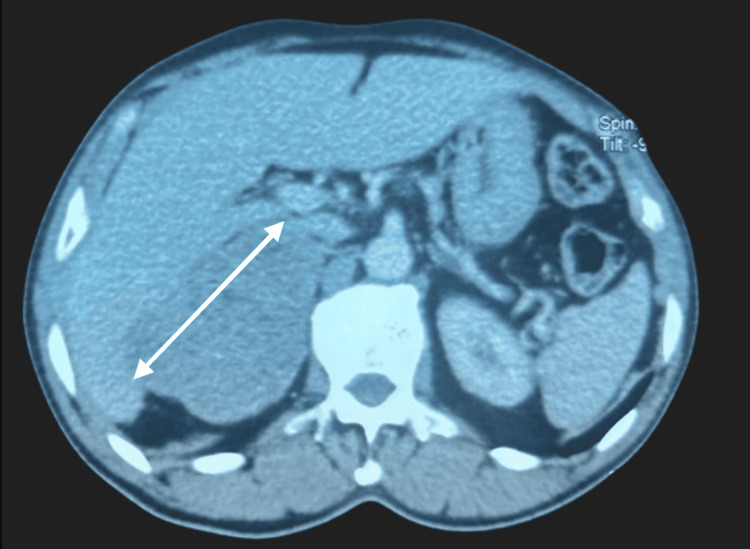
CT imaging reveals a right adrenal mass measuring 92 × 82 × 80 mm

We report the case of a 65-year-old male patient with a history of type 2 diabetes managed with metformin and well-controlled hypertension under amlodipine. The patient presented with a sensation of epigastric fullness, without Menard’s triad or other associated symptoms. Clinical examination revealed a normotensive patient with no signs of orthostatic hypotension. Abdominal CT scan revealed a large, locally infiltrative right adrenal mass measuring 92 × 82 × 80 mm (Figure 1).

**Figure 2:**
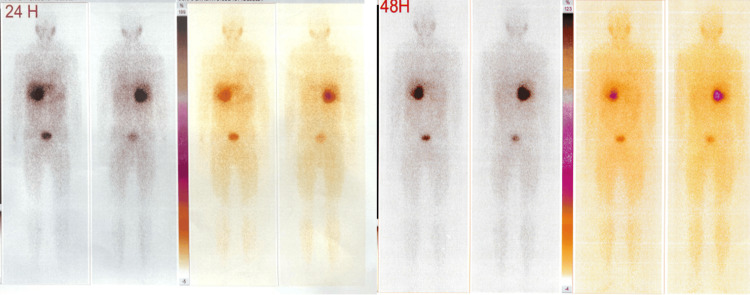
Intense and persistent MIBG uptake in the right adrenal gland at 24 hours and 48 hours, consistent with a functional right-sided pheochromocytoma without metastatic spread. MIBG: Metaiodobenzylguanidine

As part of the etiological workup, serum potassium levels and 24-hour urinary free cortisol were within normal ranges. The diagnosis of pheochromocytoma was supported by markedly elevated 24-hour urinary metanephrines at 15.04 mg/24h (reference range: 0.04-0.27 mg/24h; 55× upper limit of normal) and normetanephrines at 10.72 mg/24h (reference range: 0.07-0.46 mg/24h; 23× upper limit of normal). The iodine-123 metaiodobenzylguanidine (¹²³I-MIBG) scintigraphy revealed a strongly radiotracer-avid adrenal mass, which was the only functional imaging modality accessible in our center (Figure 2). The workup was unremarkable; blood cortisol, blood parathyroid hormone, and calcitonin levels were normal, with no evidence of multiple endocrine neoplasia.

**Figure 3:**
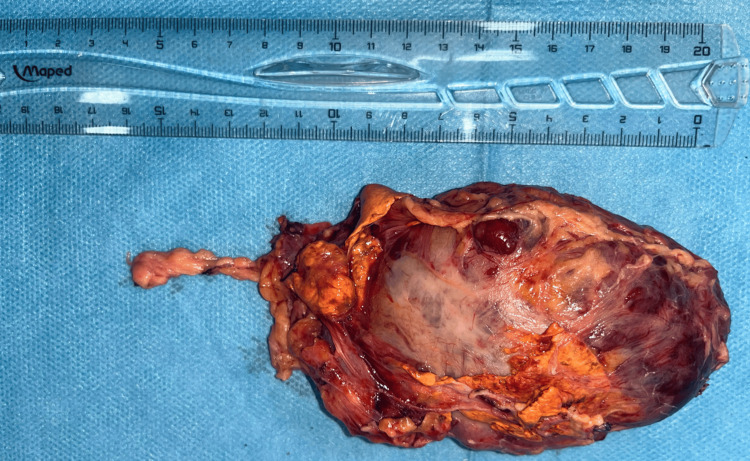
Macroscopic view of the surgical specimen

The patient underwent surgical resection through a right subcostal incision, following appropriate preoperative medical preparation. The resected specimen measured slightly over 10 cm in its largest dimension (Figure 3). Histopathological examination confirmed the diagnosis of adrenal pheochromocytoma. Genetic testing, including both germline and somatic analyses, was not performed due to limited financial resources.

**Figure 4:**
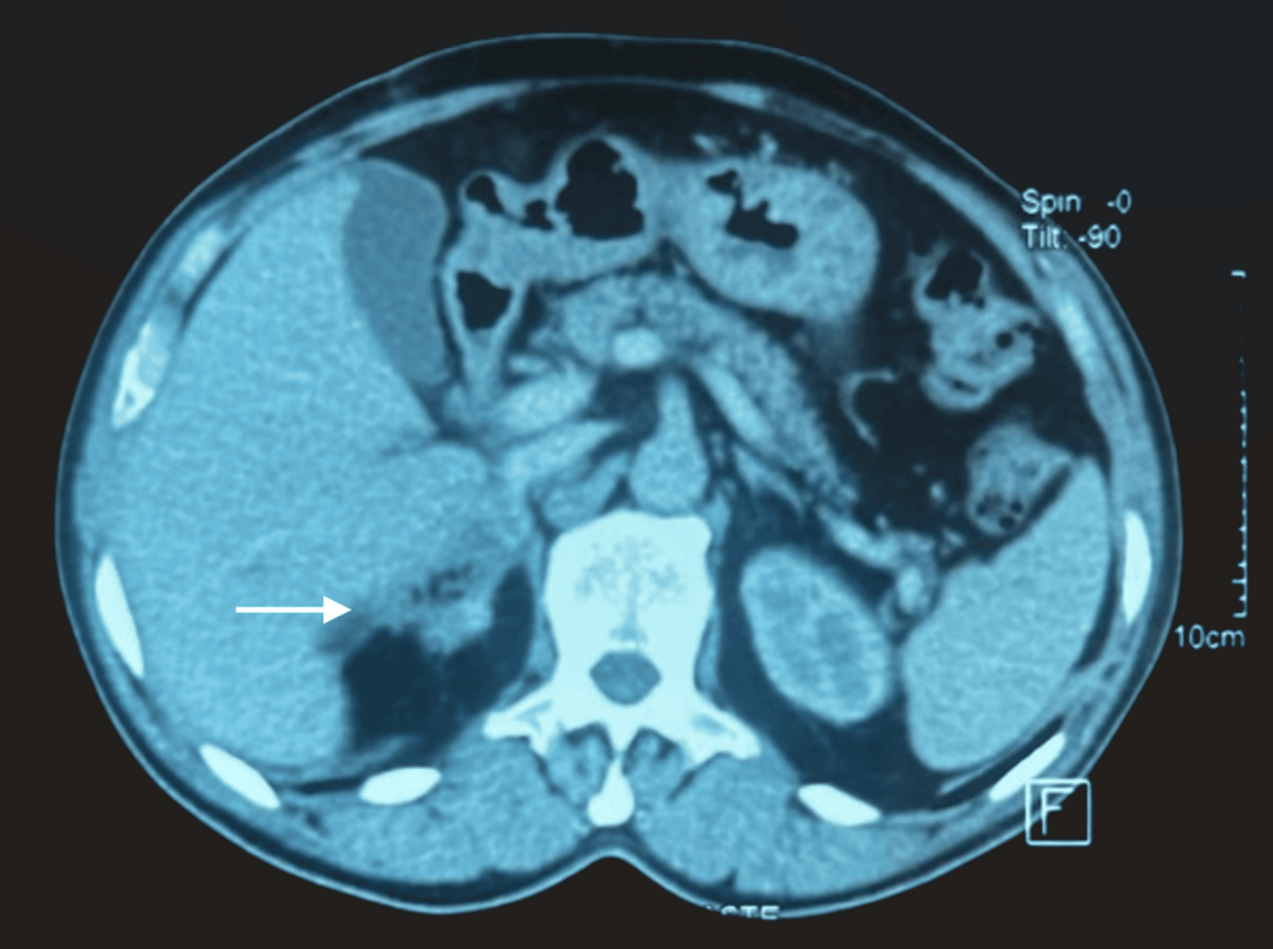
The CT imaging demonstrates a clear right adrenal surgical bed, with no evidence of residual or recurrent disease.

Postoperative recovery was uneventful. The patient remained normotensive without antihypertensive medication. Follow-up urinary methoxylated metabolites were within normal limits, with urinary metanephrine at 0.08 mg/24h and normetanephrine at 0.31 mg/24h. A follow-up abdominal CT scan showed a clear right adrenalectomy site (Figure 4).

## Discussion

Clinical manifestations of pheochromocytomas and paragangliomas depend on their secretory profile. In catecholamine-secreting tumors, symptoms result from catecholamine excess, while nonfunctioning tumors primarily cause local compressive symptoms [3]. Pheochromocytomas typically present with the classic triad of symptoms, including episodic headache, sweating, and palpitations [4]. 

However, giant lesions may paradoxically lack these symptoms. This can be attributed to factors such as tumor necrosis, a predominance of interstitial tissue over chromaffin cells, or limited catecholamine release due to encapsulation by connective tissue. As a result, serum and urinary catecholamine levels may remain within normal ranges [5-6].

Diagnosis is confirmed through biochemical tests measuring the excess production of metanephrines and catecholamines and imaging studies to identify the tumor's location. The CT scan remains the preferred imaging technique for identifying pheochromocytomas. However, when dealing with very large tumors, accurately pinpointing the originating organ may be difficult, sometimes resulting in diagnostic errors [6-7]. A meta-analysis demonstrates a sensitivity of 96% for ¹²³I-MIBG scintigraphy in non-metastatic pheochromocytoma or paraganglioma, compared to 79% in metastatic cases [8].

Surgical removal is considered the definitive treatment for both pheochromocytomas and paragangliomas. Surgical excision effectively resolves symptoms and hypertension caused by these tumors [9]. Preoperative blood pressure stabilization with alpha- and/or beta-blockers is typically required for at least 10 to 14 days to reduce the likelihood of cardiovascular events during surgery. With the rise of minimally invasive methods, laparoscopic adrenalectomy has largely replaced traditional open surgery. Nonetheless, for pheochromocytomas measuring 5 cm or larger, the choice of surgical approach should be personalized, taking into account tumor features and surgeon experience [10].

While the long-term outlook is favorable, recurrence rates within 10 years may reach up to 16%, necessitating careful monitoring. Although standardized follow-up protocols are lacking, regular evaluations, including detailed clinical assessments, blood pressure monitoring, and biochemical testing, are generally recommended every six to 12 months initially, then annually for up to a decade [11].

## Conclusions

Giant pheochromocytomas carry a high risk of serious cardiovascular complications from excessive catecholamine release. This case highlights the importance of early diagnosis, appropriate surgical management, and close follow-up to improve prognosis and contributes to the existing literature.

## References

[1] Rijken JA, Niemeijer ND, Jonker MA (2018). The penetrance of paraganglioma and pheochromocytoma in SDHB germline mutation carriers. Clin Genet.

[2] Alkaissi H, Taieb D, Lin FI, Del Rivero J, Wang K, Clifton-Bligh R, Pacak K (2025). Approach to the patient with metastatic pheochromocytoma and paraganglioma. J Clin Endocrinol Metab.

[3] Hamidi O, Young WF Jr, Iñiguez-Ariza NM (2017). Malignant pheochromocytoma and paraganglioma: 272 patients over 55 years. J Clin Endocrinol Metab.

[4] Korgali E, Dundar G, Gokce G, Kilicli F, Elagoz S, Ayan S, Gultekin EY (2014). Giant malignant pheochromocytoma with palpable rib metastases. Case Rep Urol.

[5] Li C, Chen Y, Wang W, Teng L (2012). A case of clinically silent giant right pheochromocytoma and review of literature. Can Urol Assoc J.

[6] Wang HL, Sun BZ, Xu ZJ, Lei WF, Wang XS (2015). Undiagnosed giant cystic pheochromocytoma: a case report. Oncol Lett.

[7] Ambati D, Jana K, Domes T (2014). Largest pheochromocytoma reported in Canada: a case study and literature review. Can Urol Assoc J.

[8] van der Horst-Schrivers AN, Kerstens MN, Wolffenbuttel BH (2006). Preoperative pharmacological management of phaeochromocytoma. Neth J Med.

[9] Neumann HP, Young WF Jr, Eng C (2019). Pheochromocytoma and paraganglioma. N Engl J Med.

[10] Farrugia FA, Charalampopoulos A (2019). Pheochromocytoma. Endocr Regul.

[11] Shah MH, Goldner WS, Benson AB III (2015). Neuroendocrine tumors, version 1.2015: clinical practice guidelines in Oncology. J Natl Compr Cancer Netw.

